# The Effect of Telehealth on Alzheimer's Disease, Dementia and Mild Cognitive Impairment: A Systematic Review of Clinical Trials

**DOI:** 10.1049/htl2.70065

**Published:** 2026-02-10

**Authors:** Kosar Ghaddaripouri, Reza Molavi, Sarah Montazeryan, Mohammadreza Sadegh Kharaghani, Fatemeh Taheri Soudejani, Melika Vafamand, Leila Erfannia

**Affiliations:** ^1^ Department of Health Information Management School of Health Management and Information Sciences Shiraz University of Medical Sciences Shiraz Iran; ^2^ Student Research Committee Shiraz University of Medical Sciences Shiraz Iran; ^3^ Master of Computer Engineering Torbat Heydarieh University of Medical Sciences Torbat‐e Heydarieh Iran; ^4^ Master Student of Health Information Technology Department of Health Information Management Iran University of Medical Sciences Tehran Iran; ^5^ Master Student of Medical informatics Department of Health Information Management and Medical informatics Tehran University of Medical Sciences Tehran Iran; ^6^ Department of Health Information Management Health and Human Resources Management Research Center Clinical Education Research Center Shiraz University of Medical Sciences Shiraz Iran

**Keywords:** alzheimer's disease, cognitive impairment, dementia, health care, telemedicine

## Abstract

Alzheimer's disease is a prevalent chronic condition characterised by the gradual deterioration of memory and personal abilities due to nervous system damage, requiring prolonged care and management. In contemporary healthcare, telehealth has gained recognition as an effective approach for managing chronic illnesses by improving equitable access to quality medical services and minimising expenses. The purpose of this systematic review is to evaluate the role of telehealth in enhancing the well‐being of patients with Alzheimer's disease and supporting their caregivers, as evidenced by findings from randomised controlled trials (RCTs). This systematic review concentrated on RCTs published in English, with no constraints on publication date. The search process was accomplished on 11 August, 2025, using appropriate keywords across well‐established scientific databases, including PubMed, Embase, Scopus, ScienceDirect, Web of Science and ProQuest. The quality of the studies was assessed using the Joanna Briggs Institute checklist and only those scoring above seven were included in the analysis. From an initial collection of 1242 articles, 14 trials were ultimately included in this review. Telehealth interventions demonstrated significant improvements in cognitive function, mobility and quality of life among individuals with mild cognitive impairment and Alzheimer's Disease, while also reducing caregiver burden and psychological distress. These interventions, implemented through synchronous and asynchronous delivery methods, were deemed feasible, well‐received and associated with strong adherence rates. Nonetheless, limitations such as small sample sizes and restricted access to technological resources emphasise the need for additional research to address these gaps. The findings from 13 out of 14 articles in this systematic review indicate that telehealth interventions, including virtual reality, video conferencing, computerised cognitive training and group movement programs, have the potential to significantly enhance health outcomes and quality of life for individuals with Alzheimer's disease and their caregivers compared to traditional in‐person treatments. These interventions, delivered through diverse and flexible modalities, also demonstrate cost‐effectiveness and improved caregiver well‐being, reinforcing telehealth as a scalable and effective approach for comprehensive Alzheimer's care.

## Abstract 2

The purpose of this systematic review is to evaluate the role of telehealth in enhancing the well‐being of patients with Alzheimer's disease and supporting their caregivers, as evidenced by findings from clinical trial studies.

## 1 Introduction

Alzheimer's disease is characterised by a progressive decline in cognitive abilities across various domains, leading to a loss of independence in carrying out daily tasks and impairing work performance [1, 2]. Currently, Alzheimer's disease represents a critical global health challenge, with its prevalence anticipated to rise to 75 million by 2050 [3, 4, 5]. It is ranked as the seventh leading cause of death worldwide [6]. The progression of Alzheimer's disease typically begins with a gradual decline in cognitive function and difficulties in performing everyday activities [7]. The potential contributors to Alzheimer's disease include genetic predispositions, environmental factors, lifestyle choices and age‐related changes in the brain [8]. A common early symptom of the disease is social withdrawal, which is marked by a subjective sense of loneliness and a reduction in social engagement [9, 10]. Moreover, more than half of individuals with Alzheimer's disease continue to live at home, often relying on family members for caregiving support [11, 12]. Research has demonstrated that caregivers of individuals with Alzheimer's disease experience significantly higher levels of stress, anxiety, depression and physical health challenges compared to those caring for individuals without Alzheimer's disease [13, 14].

A viable solution for supporting individuals with physical and mental health issues is the use of electronic health (eHealth) technologies [15]. The World Health Organisation defines eHealth as ‘the use of information and communication technologies (ICT) for health’ [16]. Telehealth, a prominent tool within eHealth, involves the transmission of medical data over various distances through communication technologies to deliver healthcare services [17]. Once considered a concept for the future, telehealth is now an integral part of contemporary healthcare practices [18]. Recent advancements in medical technology have led to the widespread use of telehealth applications, including electronic consultations, telephone triage, post‐operative follow‐ups and mental health services [19]. Evidence indicates that telehealth improves symptom management and enhances satisfaction among patients and their families [20, 21]. Additionally, research by Yue Ma and colleagues highlighted telehealth as a valuable tool in managing chronic disease patients, proposing it as an effective strategy to promote equitable access to quality healthcare and reduce overall costs [22, 23].

In recent years, increased recognition of the challenges associated with Alzheimer's disease has led to the development of innovative solutions aimed at improving the quality of life for those affected [24]. Telehealth enables individuals with Alzheimer's disease to overcome transportation difficulties, providing them with access to diagnoses and care remotely [25]. Furthermore, the growing demand for healthcare facilities, particularly in densely populated or underserved areas, has created significant barriers to timely patient care [26]. Telehealth can help address these challenges by reducing waiting times and accelerating patient assessments [27]. As a result, mobile health programs offering psychological support present a promising solution for enhancing treatment access for individuals who are unable to attend in‐person appointments [28].

Several studies have investigated the effects of telehealth on patients with Alzheimer's disease. In a systematic review, Cotelli and colleagues compared the effectiveness of cognitive telerehabilitation to face‐to‐face rehabilitation for patients with mild cognitive impairment, Alzheimer's disease and frontotemporal Alzheimer's disease. Their results indicated that cognitive telerehabilitation has significantly positive effects on patients with neurological diseases compared to traditional in‐person treatment [29]. Similarly, Christie and colleagues conducted a systematic review on eHealth interventions for informal caregivers of individuals with Alzheimer's disease. They found that such interventions led to improvements in caregivers' lives and enhanced their caregiving skills [30].

In a systematic review by Gillam and colleagues, which focused on identifying critical factors for implementing eHealth in individuals with Alzheimer's disease, it was highlighted that effective implementation strategies should take into account both the needs of the end‐users and the characteristics of the care environment [31]. Moreover, deep learning techniques offer significant potential for enhancing the diagnosis and risk assessment of mental disorders, including Alzheimer's disease, thereby providing a valuable complement to telehealth interventions [32]. Furthermore, a study by Kruse and colleagues, which examined the effectiveness of telehealth interventions for memory care in Alzheimer's disease patients, found that telehealth improves memory, cognition, brain activity, quality of life and reduces depression. The study identified key factors contributing to the acceptance of telehealth, including education, time efficiency, cost‐effectiveness and low reimbursement rates [33].

In a systematic review by Lorito and colleagues, the effectiveness of digital health interventions on cognitive, behavioural, physical, psychological and daily activity outcomes in individuals with Alzheimer's disease and mild cognitive impairment (MCI) was assessed. They found that a combination of remote and in‐person treatment could help reduce costs while enhancing effectiveness [34]. However, randomised controlled trials (RCTs) are widely regarded as providing the strongest evidence in any research field [35]. RCTs are valuable prospective studies that assess the efficacy of new treatments or interventions [36]. Despite the existence of RCTs examining telehealth in Alzheimer's disease, no systematic review had previously synthesised this evidence. Therefore, this review aimed to evaluate the effects of telehealth on clinical outcomes in patients with Alzheimer's disease and on the well‐being of their caregivers.

## 2 Methods

### 2.1 Study Design

As noted by El‐Gazzar, systematic reviews play an important role in synthesising existing evidence, identifying areas that require further investigation and thereby contributing to the creation of new knowledge. In line with this, the present review applies this rigorous process within the context of telehealth for Alzheimer's disease [37]. The purpose of systematic reviews in the healthcare field is to gather, assess and synthesise findings from all relevant studies on a particular health topic, thus providing decision‐makers with comprehensive and accessible evidence [38]. The increasing number of health journals has made it demanding for healthcare decision‐makers to discuss all current evidence for an evidence‐based approach [39]. This systematic review was conducted following the PRISMA 2020 guidelines to guarantee the precise and reliable presentation of the findings from the included studies [40, 41].

The methodology employed in this study adheres to the PRISMA statement guidelines and consists of five key steps: (1) literature review, (2) inclusion and exclusion criteria, (3) study selection, (4) quality appraisal and (5) data extraction and synthesis [42].

### 2.2 Literature Review: Databases and Keywords

A literature search was performed on 11 August, 2025, across several databases, including PubMed, Embase, Scopus, ScienceDirect, Web of Science and ProQuest. The search utilised MeSH and Emtree terms, with the following keywords and phrases: (telehealth OR telemedicine OR ehealth) AND (Dementia OR Alzheimer OR cognitive impairment) AND (effectiveness OR physical activity OR cognitive function).

### 2.3 Inclusion and Exclusion Criteria

Inclusion Criteria:

The studies selected met the following criteria:

1. Full‐text availability.
2. Publication in English only.
3. Focus on telehealth in Alzheimer's disease, dementia, or MCI.
4. Clinical trial design.
5. Publication date up to 11 August, 2025.

Exclusion Criteria

The following studies were excluded:

1. Studies where full‐text access was not available.
2. Non‐English language studies.
3. Review articles, letters, dissertations, technical notes, posters and studies applying telehealth to fields unrelated to Alzheimer's disease.

### 2.4 Study Selection

**FIGURE 1:**
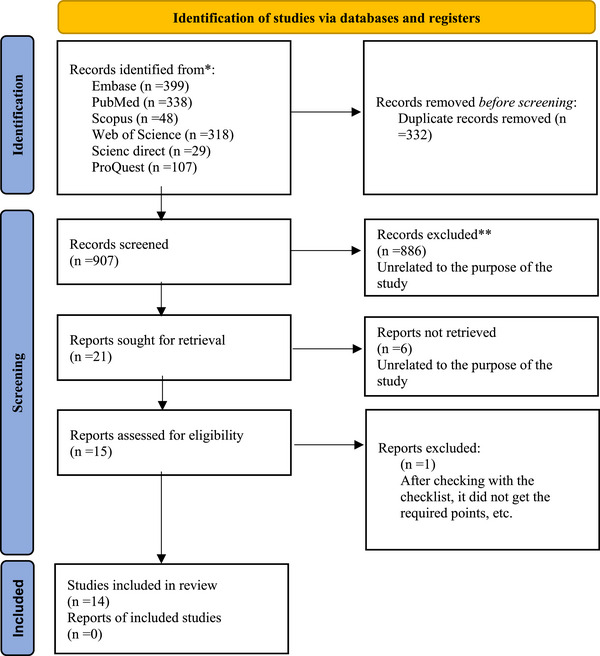
The details of the article selection process.

A total of 1242 articles were identified initially. After removing 332 duplicates, 907 articles remained. Upon reviewing the titles and abstracts, 886 articles were excluded. The full texts of the remaining 21 articles were assessed based on the inclusion and exclusion criteria, resulting in the exclusion of 6 articles. No additional records were identified through manual searching of reference lists or other sources. The details of the article selection process are illustrated in Figure 1.

### 2.5 Quality Appraisal

The quality of the studies contained in this study was evaluated utilising the Joanna Briggs Institute (JBI) quality evaluation checklist for RCTs [43]. This checklist contains 13 questions aimed at evaluating study quality. A score of 1 was given for each ‘yes’ answer and a score of 0 for each ‘no’ answer, with the highest possible score being 13. Studies scoring less than 7 were excluded from the review. The quality appraisal results, summarised in Table 1, show that no significant bias was present in the studies and only one study did not encounter the essential score [44]. The JBI checklist is supplied in Appendix 1.

**TABLE 1:** Summary of quality evaluation using JBI evaluation checklist.

Study	Q1	Q2	Q3	Q4	Q5	Q6	Q7	Q8	Q9	Q10	Q11	Q12	Q13	Score
Sara Bernin et al., 2023. [45]	Y	Y	Y	N	N	N	N	Y	Y	Y	Y	Y	Y	9
Rosa Manenti et al., 2020. [46]	Y	Y	Y	N	N	Y	Y	Y	Y	Y	Y	Y	Y	11
Fuzhong Li et al., 2021. [47]	Y	Y	Y	N	N	Y	Y	Y	Y	Y	Y	Y	Y	11
Mariam Torkamani et al., 2014. [48]	Y	N	N	N	N	N	N	Y	Y	Y	Y	Y	Y	7
Clarissa A et al., 2021. [49]	Y	N	Y	N	N	N	Y	Y	Y	Y	Y	Y	Y	9
Kübra Nur Menengi˙ç et al., 2022. [50]	Y	N	Y	N	N	N	Y	Y	Y	Y	Y	Y	Y	9
Rachel L Burton et al., 2018. [51]	Y	Y	Y	N	N	N	Y	Y	Y	Y	Y	Y	Y	10
Rose‐Marie Dröes et al., 2019. [52]	Y	N	Y	N	Y	N	Y	Y	Y	Y	Y	Y	Y	10
Elizabeth K. Rhodus et al., 2023. [53]	Y	N	Y	N	N	Y	N	N	Y	Y	Y	Y	Y	8
David P. Neal et al., 2023. [54]	Y	N	Y	N	N	N	Y	Y	Y	Y	Y	Y	Y	9
Nicola Coley et al., 2022. [55]	Y	N	Y	N	N	N	Y	Y	Y	Y	Y	Y	Y	9
Julia Zuschnegg et al., 2025 [56]	Y	Y	Y	N	N	Y	Y	Y	Y	Y	Y	Y	Y	11
Deborah E. Barnes et al., 2025 [57]	Y	Y	Y	N	N	Y	Y	Y	Y	Y	Y	Y	Y	11
Graessel et al., 2024 [58]	Y	Y	Y	Y	N	Y	Y	Y	Y	Y	Y	Y	Y	12

### 2.6 Data Extraction and Synthesis

Following study selection and quality assessment, data were extracted from the included articles using a pre‐designed form. This form captured key study characteristics, including authors, country of publication, year, type of technology used, telehealth modality, type of intervention, number and mean age of participants, target population, intervention duration, telehealth settings, scales/tests employed, assessment time points and clinical outcomes. Extracted data were then synthesised to compare and contrast findings across studies.

## 3 Result

The characteristics of the 14 included studies are presented in Table 2. Four studies (29%) were conducted in the United States [47, 49, 53, 57], two (14%) in Italy [45, 46] and two (14%) in the Netherlands [52, 54]. The remaining studies were carried out in Turkey [50], Canada [51], Austria [56], Germany [58], a multicenter study across France, Finland and the Netherlands [55] and another across the United Kingdom, Spain and Greece [49]. In terms of publication year, two studies were published in 2025 [56, 57], two studies were published in 2023 [45, 53] and two in 2021 [47, 49], while one study each was published in 2024 [58], 2022 [50], 2020 [46], 2019 [52], 2018 [51] and 2014 [48]. The included studies evaluated a total of 3739 participants, with sample sizes ranging from 6 to 2724. The mean number of participants per study was 267 (SD = 710.8), while the median was 58 (IQR = 30–117), indicating that most studies were relatively small in scale, with one large multicentre trial substantially increasing the overall mean. The design of input studies includes: RCT [45, 50], multicentre rater‐blinded, active RCT [46], single‐blinded, randomised, parallel‐group feasibility trial [47], multi‐centre RCT [48, 49], small‐scale RCT [51], explorative RCT [52], single‐blind, three‐arm, parallel RCT [53], non‐blinded, RCT [54], parallel group RCT [55], two‐arm, parallel group randomised controlled trial (RCT) with a longitudinal approach [56], randomised controlled trial (RCT) with a waitlist control group [57] and prospective double‐blind randomised controlled intervention [58].

**TABLE 2:** Summary of the included studies.

Study	Country	Study design, sample size	Participants	Inclusion criteria	Exclusion criteria
Sara Bernin et al., 2023. [45]	Italy	RCT **Sample size**: 56 patients (telerehabilitation: 31 and in‐person rehabilitation: 25) female: 30 male: 26	**mean age**: 72.78 People with SCD, mNCD and MNCD due to Alzheimer's disease	Age over 50 years; more than 5 years of formal education; Diagnosis of SCD, mNCD, or MNCD (due to Alzheimer's disease or vascular dementia); A CDR score between 0 and 1; An MMSE raw score of 20 or higher.	(a) Cognitive deficits resulting from acute or systemic medical conditions (e.g., traumatic brain injury or intracranial neoplasm); (b) clinically significant neuropsychiatric disorders, including severe mood or behavioural disturbances; (c) marked sensory impairments (such as profound deafness or blindness) or deficits in motor function of the dominant upper limb.
Rosa Manenti et al., 2020. [46]	Italy	Multicentre rater‐blinded, active‐controlled and randomised study **Sample size**: 49 patients (the clinic‐VRRS + Tele@H‐VRRS group: 18 patients clinic‐VRRS + Tele@H‐UCS group: 14 patients clinic‐TAU group: 17 patients	People with MCI	a) Memory criticisms; (b) protection of general cognitive functioning established by MMSE scores from 24 to 30; (c) age > 65 years; (d) global CDR score of 0.5; (e) protection of functional activities; (f) lack of measures for a diagnosis of dementia according to DSM‐V; and (g) lack of mood and anxiety disorders.	(a) Other previous or current neurological or major psychiatric disorders; (b) visual perception disorder and/or hearing loss; (c) history of traumatic brain injury, brain tumour, or stroke; and (d) history of alcohol abuse. None of the participants had experienced cognitive training protocols within the year before registration or during the whole period of the current study (from baseline to the last follow‐up assessment).
Fuzhong Li et al., 2021. [47]	United States	Single‐blinded, randomised, parallel‐group feasibility trial **Sample size**: 30 patients (intervention: 15 control: 15)	**Mean age**: 76.2 years People with MCI	(a) Were over 65 years of age; (b) reported memory loss, as corroborated by an informant; (c) attained a clinical dementia rating (CDR) score of 0.5 or less and a mini‐mental state examination (MMSE) score of 24 or higher; (d) had not engaged in any structured, rigorous physical activity or exercise program in the previous three months; and (e) obtained medical clearance from a healthcare provider.	(a) Individuals who were unable to adhere to the 24‐week study period; (b) those exhibiting physical frailty or impaired ambulation; (c) those unwilling to undergo group assignment.
Mariam Torkamani et al., 2014. [48]	United Kingdom	A multi‐centre randomised controlled pilot study **Sample size**: 60 (intervention: 30 control: 30)	**Mean age**: 78.03 patients and their carers with dementia	Community‐dwelling participants with continuous full‐time caregiving support exhibited a Barthel index score exceeding 35 (reflecting a degree of functional independence) and demonstrated a mini‐mental State examination score between 9 and 21 (indicative of moderate to mild cognitive impairment).	Not mentioned
Clarissa A et al., 2021. [49]	United States	Multi‐site RCT **Sample size**: 124 **Patients**: 56 (intervention: 28, control: 28) **Caregiver**: 68 (intervention: 31, control: 37)	Mean age of patients: 76.1 Patients with dementia and their family caregivers	Individuals with dementia who were community‐dwelling and had been diagnosed with dementia at a severity level of mild or greater, as determined by the FAST scale.	Individuals with dementia who presented with a documented diagnosis of Huntington's disease, schizophrenia, bipolar disorder, significant hearing impairment and/or intellectual disability.
Kübra Nur Menengi˙ç et al., 2022. [50]	Turkey	Pilot RCT **Sample size**: 20 (intervention: 10, control: 10)	Mean age of patients: 79.15 Patients with AD	(a) Age > 65 years, (b) diagnosis of AD according to the NINCDSADRDA criteria, (c) Mini‐MMSE of 13–24, (d) CDR score 1–2, (e) standard use and regular doses of cholinesterase inhibitors and/or memantine for at least a month, (f) sufficient communication skills to comprehend the teachings and (g) and living with a keeper who could use technological tools.	(a) Dementia kinds other than AD; (b) pulmonary, neurologic, musculoskeletal, or rheumatologic disease that might prevent exercise; (c) dangerous medical condition (e.g. uncontrolled diabetes or hypertension, deep vein thrombosis); (d) having standard rehabilitation assistance from an establishment or person; (e) standard exercise habits; (f) visual or auditory deficits or behavioural problems that would make communication difficult; and (g) who had difficulties in adjusting to online applications and who moved to a different city or home from the time they were included in the study.
Rachel L Burton et al., 2018. [51]	Canada	Small‐scale RCT **Sample size**: 6 (intervention: 3, control: 3)	Mean of age: 71.83 patients with SCI and no diagnosis, MCI, early‐stage dementia due to AD, or mixed AD and vascular dementia	Individuals exhibiting subjective cognitive impairment without a formal diagnosis, mild cognitive impairment, early‐stage Alzheimer's disease, or a concomitant diagnosis of Alzheimer's and vascular dementia	Not mentioned
Rose‐Marie Dröes et al., 2019. [52]	Netherlands	Explorative RCT **Sample size**: 282 (DemenTalent iMCSP: 39 regular MCSP: 54 No day care/support: 189)	People with dementia and caregivers	Not mentioned	Not mentioned
Elizabeth K. Rhodus et al., 2023. [53]	United States	Single‐blind, three‐arm, parallel, RCT **Sample size**: 30 (HARMONY: 10, standardised: 10, control: 10)	Mean of age: 77.9 People with dementia and their care partners	(a) A CDR scale score of 1.0 or higher; (b) residence in the community and caregiver‐identified behavioural disturbances; (c) a stable medical condition; (d) consistent medication use for at least one month prior to screening; (e) no involvement in cognitive rehabilitation or occupational therapy programs; (f) the availability of a willing care partner.	Confinement to bed; physically aggressive behaviour; severe or complete sensory impairments; a diagnosis of a major psychiatric disorder; and recent use of investigational medications within 30 days prior to screening.
David P. Neal et al., 2023. [54]	Netherlands	A non‐blinded RCT **Sample size**: 150 (intervention: 76, control: 74)	People with dementia and their caregivers	Not mentioned	Not mentioned
Nicola Coley et al., 2022. [55]	France	parallel group RCT **Sample size**: 2724 (intervention: 1389, control: 1335)	Not mentioned	(a) Community‐dwelling individuals without dementia, (b) aged 65 years or older; (c) possessed at least basic computer literacy; (d) had either two or more CVD risk factors (such as hypertension, dyslipidemia, overweight, smoking, or physical inactivity) or a documented history of CVD or diabetes.	Not mentioned
Julia Zuschnegg et al., 2025 [56]	Austria	Two‐arm, parallel group RCT with a single‐blinded design and longitudinal approach **Sample size**: 22 (intervention: 11, control: 11)	Community‐dwelling people with mild to moderate AD, living at home in Austria, with an available informal caregiver willing to participate; mean age ∼76; sex: 8F/3 M per group	(1) Confirmed diagnosis of AD; (2) age over 40 years; (3) stable dementia medication for at least 3 months; (4) fluent in German; (5) sufficient physical, auditory and visual skills to perform a tablet‐based multimodal training; (6) an available informal caregiver willing to participate in the study alongside the participant	(1) Participating in another clinical trial; (2) prior history of cancer, myocardial infarction, hepatitis, HIV, syphilis, or current depressive episode; (3) signs in MRI such as lacunar infarction, confluent lesions, or other focal lesions
Deborah E. Barnes et al., 2024 [57]	USA	Randomised controlled trial with a waitlist control group. **Sample size**: 97 dyads (intervention (moving together): 54 dyads, control (waitlist): 43 dyads)	Community‐dwelling dyads consisting of people with cognitive impairment (PWCI: mean age 76 ± 11 years, 43% female, 80% non‐Hispanic White) and their care partners (CPs: mean age 66 ± 12 years, 78% female, 71% non‐Hispanic White); PWCI had mild symptom severity related to Alzheimer's disease and related dementias	English‐language proficiency; U.S. residency; access to a device with a video camera enabling participation in two‐way, livestreaming video classes; mild symptom severity for PWCI (defined as quick dementia rating system score of 2.5 to 12.5); CPs willing to participate in online classes with PWCI from the same physical location and to answer study questionnaires	Not explicitly listed; inferred as failure to meet inclusion criteria (e.g., non‐English speakers, non‐U.S. residents, lack of required technology, moderate or severe symptom severity in PWCI, or unwillingness of CPs to participate as described)
Graessel et al., 2024 [58]	Germany	Prospective double‐blind RCT, conducted entirely virtually **Sample size**: 89 (intervention: 44, control: 45)	Community‐dwelling people aged 60 + with psychometrically diagnosed MCI, living in Bavaria, Germany	Experiencing subjective cognitive decline with psychometric MCI diagnosis (MoCA score ≤ 24 and MMSE score ≥ 24); (2) ownership of a digital device; (3) age at least 60 years; (4) signed informed consent form	(1) Technical requirements not met; (2) MoCA score indicating no cognitive impairment (MoCA >24); (3) MMSE score indicating dementia (MMSE 12), or other psychiatric or neurological disorders associated with cognitive decline

**Abbreviations**: BI, Barthel index; CDR, clinical dementia rating; CVD, cardiovascular disease; FAST, functional assessment staging; HARMONY, helping older adults create and manage occupations successfully; MCI, mild cognitive impairment; MMSE, mini‐mental state examination; MNCD, major neurocognitive disorder; mNCD, mild neurocognitive disorder; MRI, magnetic resonance imaging; NINCDSADRDA, National Institute of Neurological and Communicative Disorders and Stroke and Alzheimer's disease; SCD, subjective cognitive decline.

### 3.1 Characteristics of the Telehealth Interventions

Table 3 provides a summary of the telehealth interventions. The majority of studies employed synchronous (real‐time) feedback, while one study employed asynchronous (off‐line) feedback [46] and three studies incorporated a combination of both methods [48, 54, 55]. Various telehealth platforms were used, including Zoom in two studies [45, 52], HomeCoRe and CoRe in one study [45], Khymeia in one study [46], ALADDIN in one study [48], STAR e‐learning in one study [52], FindMyApps in one study [54] and Vital Health Software in one study. Additionally, three studies [56, 57, 58] introduced other platforms or approaches, further diversifying the technological modalities used. The interventions lasted from 6 weeks to 18 months. Among the 14 studies, 8 (57%) were fully conducted remotely at the patients’ homes [47, 48, 50, 51, 52, 53, 54, 55], while 6 studies (43%) adopted a hybrid model that integrated both home and hospital/clinic settings [45, 46, 49, 56, 57, 58]. One study specifically explored the feasibility of using Zoom technology for individuals with MCI over 24 weeks [47].

**TABLE 3:** Summary of telehealth interventions in the included studies.

Study	Type of technology	Modality of telehealth	Type of telehealth intervention	Study goal	Clinical outcomes	Duration of intervention	Telehealth settings
Sara Bernin et al., 2023. [45]	**Hardware**: Laptop, desktop PC **Software**: Internet connection, HomeCoRe and CoRe, Zenodo	Synchronous (internet‐delivered).	**Telehealth**: The HomeCoRe research software, installed on the participant's personal laptop, was utilised to deliver remote care. **Conventional Intervention**: In‐person treatment was provided in a hospital setting, incorporating the CoRe application on a laptop or desktop computer.	to identify factors associated with the preference for either tele‐rehabilitation (TR) or in‐person cognitive therapy (CT) programs in older adults at risk of dementia or with early cognitive impairment.	Participants who selected the TR modality of CT exhibited significantly higher levels of cognitive reserve and adopted more protective lifestyle habits, such as regular physical activity and following a Mediterranean diet, compared to those who preferred in‐person CT.	**Telehealth**: 6‐week program (3 sessions/week, each lasting approximately 45 min) **Conventional intervention**: similar to tele‐rehabilitation	Home, hospital setting
Rosa Manenti et al., 2020. [46]	**Hardware**: VR, Tablets **Software**: Khymeia	combined: synchronous and asynchronous (internet‐delivered, videoconference)	**Telehealth**: In this study, participants were divided into three groups: **1. Group A (clinic‐VRRS + Tele@H‐VRRS)**: Participants first received face‐to‐face cognitive virtual reality rehabilitation (VRRS) with 12 individualised sessions over 4 weeks, followed by home‐based cognitive VRRS training (36 sessions, 3 times a week) through telehealth. **2. Group B (clinic‐VRRS + Tele@H‐UCS)**: Participants received face‐to‐face cognitive VRRS treatment followed by 36 sessions of home‐based unstructured cognitive stimulation (3 sessions per week) via telehealth.	This study assesses the efficacy of face‐to‐face cognitive VRRS. It compares it with conventional face‐to‐face cognitive therapy as typical (TAU) for individuals with MCI.	1. A powerful improvement in cognitive abilities, including memory, language and visuo‐constructional abilities, was regarded after the end of the face‐to‐face cognitive VRRS cure when compared to the face‐to‐face cognitive cure as usual (TAU). 2‐ The results show that cognitive rehabilitation using VRRS is totally optimistic, **3.Conventional intervention (clinic‐TAU)**: Participants received the standard face‐to‐face cognitive treatment at the clinic. These groups were compared to assess the effectiveness of combining face‐to‐face and telehealth‐based cognitive rehabilitation interventions in enhancing cognitive function in individuals with dementia or cognitive impairments.	**Telehealth**: 17‐week program (48 sessions) **Conventional intervention**: 4‐week 12 sessions as usual	Home, Clinic
Fuzhong Li et al., 2021. [47]	**Hardware**: Phone **Software**: Internet connection, Zoom	Synchronous (internet‐delivered, video calls).	**Telehealth**: interventions were delivered online via Zoom **Conventional intervention**: Tai Ji Quan, Stretching exercises	to evaluate the feasibility of delivering a virtual (online) falls prevention intervention for older adults diagnosed with MCI.	1‐. A Tai Ji Quan‐based falls prevention intervention was successfully implemented for community‐dwelling older adults with MCI through Zoom technology. 2‐The 24‐week intervention showed good program fidelity, with high participant compliance and a low rate of attrition. 3. Participants did not report major complaints during the virtual classes and no significant adverse events related to the intervention occurred throughout the feasibility trial.	**Telehealth**: 24‐week program (48 sessions, each lasting approximately 60 min) **Conventional intervention**: similar to tele‐rehabilitation	Home
Mariam Torkamani et al., 2014. [48]	**Hardware**: Laptops **Software**: Internet connection, ALADDIN, SPSS software for Windows (version 20)	Combined: synchronous and Asynchronous (internet delivered, built‐in messenger)	**Telehealth**: The intervention was implemented through a digital platform called ALADDIN, which encompasses four primary features: ‘ALADDIN TV’, ‘social networking’, ‘my tasks’ and ‘contact us’.	To explore whether providing access to information and support through a telemedicine system, along with frequent monitoring of people with dementia (PwD) and their caregivers, could alleviate caregiver burden and distress while enhancing the caregiver's quality of life (QoL).	1‐The findings indicated a reduction in caregiver burden and distress, along with improvements in the QoL of caregivers who had access to the ALADDIN computerised platform. 2‐Throughout the study period and at the final assessment, participants in the platform group demonstrated more significant improvements compared to the control group. 3‐Caregivers using ALADDIN observed a more pronounced reduction in burden during the study period compared to those in the control group and their overall response to using ALADDIN was positive. 4‐The results revealed a significant interaction effect on caregiver QoL. 5‐Frequent patient monitoring proved valuable in providing crucial clinical data that helped improve the management of the person with dementia's care, including facilitating referrals and communication with other healthcare professionals when needed.	**Telehealth**: 6 months **Conventional intervention**: similar to tele rehabilitation	Home
Clarissa A et al., 2021. [49]	**Hardware**: Any device that can connect to internet **Software**: Video recordings	Synchronous (internet delivered).	**Telehealth**: Care delivery involved video recordings utilising the FamTechCare platform. **Conventional intervention**: Support was provided via telephone at two designated research sites.	to assess the cost‐effectiveness of the FamTechCare intervention in comparison to standard telephone‐based support, using caregiver retrospective recall for evaluation.	The FamTechCare intervention has shown notable decreases in caregiver depression and improvements in caregiver competence, relative to standard telephone‐based support.	**Telehealth**: 12 weeks **Conventional intervention**: similar to tele‐rehabilitation	Home, clinic
Kübra Nur Menengi˙ç et al., 2022. [50]	**Hardware**: computer or tablet **Software**: Internet connection, video conference program	Synchronous (internet‐delivered).	**Telehealth**: software‐integrated videoconferencing with screen‐sharing and participants conducted computer‐based actions	To explore the effectiveness of training care via home‐based TR in AD.	1‐Real‐time motor‐cognitive dual‐task exercise via telehealth may improve cognition, practical mobility and independence and decrease anxiety and depression symptoms in individuals with early to middle‐stage AD, while also enhancing caretaker well‐being. 2‐Real‐time supervision appears to be a safe procedure when performed at home with caregiver guidance, with synchronised cure showing primary pedagogy for patients with cognitive impairments. 3‐ A 6‐week online motor‐cognitive dual‐task exercise program can result in significant modifications in cognitive function, mobility, daily living activities, independence, depression, anxiety and caregiver well‐being when compared to no intervention.	**Telehealth**: 6 weeks **Conventional intervention**: similar to tele rehabilitation	Home
Rachel L Burton et al., 2018. [51]	**Hardware**: Any device that can connect to internet **Software**: Internet connection, video conference program	Synchronous (internet‐delivered).	**Telehealth**: home‐based intervention via video conferencing	To explore the acceptability and feasibility of providing cognitive rehabilitation to individuals diagnosed with Alzheimer's disease‐related dementia through telehealth videoconferencing.	Cognitive rehabilitation can be successfully modified for delivery via telehealth videoconferencing for older adults experiencing both subjective and objective memory impairments.	**Telehealth**: 8 weeks, **Conventional intervention**: similar to tele‐rehabilitation	Home
Rose‐Marie Dröes et al., 2019. [52]	**Hardware**: telephone **Software**: STAR e‐Learning course	Synchronous (internet‐delivered).	**Telehealth**: Dementelcoach (telephone‐based coaching) and the STAR e‐Learning program designed for caregivers. **Conventional intervention**: Individualised meeting centres support program (iMCSP), which incorporates DemenTalent, a program tailored for individuals living with dementia.	Examined the effectiveness of the integrated multi‐component support program (iMCSP), which includes DemenTalent (a program where individuals with dementia volunteer based on their talents), Dementelcoach (telephone coaching) and STAR e‐learning for caregivers, in comparison to the regular multi‐component support program (MCSP) and the no day care support.	Caregivers in both the DemenTalent group and the Dementelcoach/STAR group reported higher levels of happiness compared to caregivers of individuals with dementia who did not receive any support.	**Telehealth**: 6 months **Conventional intervention**: similar to tele rehabilitation	Home
Elizabeth K. Rhodus et al., 2023. [53]	**Hardware**: phone **Software**: online survey format, video conferencing, telehealth application, Zoom	Synchronous (internet‐delivered, telephone video calls).	**Telehealth**: utilisation of video conferencing technologies **Conventional intervention**: innovative occupational therapy telehealth program, designated as HARMONY (helping older adults create and manage occupations successfully)	to evaluate the feasibility of the HARMONY intervention, focusing on its delivery to community‐dwelling older adults with Alzheimer's disease (AD) and their caregivers through telehealth, specifically via videoconferencing methods.	1‐The individualised sensory‐based intervention can be effectively delivered through telehealth and demonstrates positive impacts on the performance and behavioural symptoms of individuals with Alzheimer's disease (AD).’ 2‐Improvements were noted in the severity of behavioural symptoms and the distress experienced by caregivers.’	**Telehealth**: 10 weeks, **Conventional intervention**: similar to tele‐rehabilitation	Home
David P. Neal et al., 2023. [54]	**Hardware**: A tablet (running iPadOS or Android) **Software**: the FindMyApps app, the app database,	combined: synchronous and asynchronous (internet‐delivered, video conference)	**Telehealth**: a tablet‐based eHealth intervention	To compare the effects of the FindMyApps intervention with a control intervention (standard digital care) on social participation and self‐management in individuals with dementia or MCI after a 3‐month period.	A trend was observed indicating that arm participants in the experimental group downloaded more apps, although no statistically significant differences were found between the experimental and control arm groups in terms of the overall frequency of tablet usage.	**Telehealth**: 3 months **Conventional intervention**: similar to tele rehabilitation	Home
Nicola Coley et al., 2022. [55]	**Hardware**: computer **Software**: Internet, Vital Health Software	combined: synchronous and Asynchronous (internet‐delivered, built‐in messenger)	**Telehealth**: An internet‐based platform offering remote guidance from a lifestyle coach, facilitated through a computer messaging system, with technical development conducted by Vital Health Software. **Conventional intervention**: A basic static internet platform that solely provides fundamental health information without any coaching support.	to describe the engagement of older adults with an eHealth intervention, identify factors that influence their participation and investigate the associations between engagement levels and changes in cardiovascular and dementia risk factors, such as blood pressure, cholesterol levels, BMI, physical activity, diet and overall risk scores for cardiovascular and dementia conditions.	1. An eHealth lifestyle intervention led to notable improvements in risk factors among older adults. 2. Engaging older adults in an eHealth self‐management intervention for lifestyle changes is practical, with higher engagement levels resulting in more significant improvements in dementia‐related biological and behavioural risk factors, as well as cardiovascular health.	**Telehealth**: 18 months **Conventional intervention**: similar to tele‐rehabilitation	Home
Julia Zuschnegg et al., 2025 [56]	**Hardware**: Tablet **Software**: custom‐developed multimodal training app (cognitive + physical exercises)	Home‐based digital training via tablet; weekly in‐person supervised sessions; telephone hotline support.	**Telehealth**: Tablet‐based multimodal training (cognitive + physical exercises, weekly supervised, self‐practice). **Conventional intervention**: paper‐and‐pencil exercises (unsupervised, quarterly delivery).	Evaluate if tablet‐based multimodal training stabilises cognition and reduces brain atrophy in AD compared to paper‐based cognitive exercises.	The intervention group showed stable global cognitive function (MMSE score), while the control group experienced a decline (significant time‐by‐group interaction: F1,14 = 5.083, *p* = .041). No significant differences in other cognitive domains (e.g., memory, attention, executive functions and verbal fluency) or cerebral volumes (e.g., hippocampal volume). No measurable transferability to brain structure.	**Telehealth**: 6 months (tablet training + weekly supervised visits). **Conventional intervention**: 6 months (paper‐and‐pencil exercises, quarterly delivery). (Originally planned 18 months, shortened due to COVID‐19).	Home‐based setting; Tablet use; Weekly supervised sessions by Red Cross staff; caregiver involvement; telephone hotline support; neuropsychological + MRI assessments at hospital (baseline and 6 months).
Deborah E. Barnes et al., 2024 [57]	**Hardware**: computer, tablet, or device with a video camera and internet access **Software**: Online videoconferencing platform for livestream classes (Moving Together program)	Synchronous telehealth, involving real‐time, interactive livestream group sessions.	**Telehealth (intervention)**: Online, livestream, mind‐body, group movement program (‘Moving Together’), 1 h, 2 days/week for 12 weeks **Conventional intervention (control)**: Waitlist control (usual activities, then later received the same program)	To determine whether the Moving Together program improves quality of life (QOL) in people with cognitive impairment (PWCI) or care partners (CPs) and to explore potential mechanisms of action.	PWCI reported significantly improved self‐rated QOL (effect size [ES] = 0.474, *p* = 0.048 from baseline to 12 weeks compared to waitlist; ES = 0.663, *p* = 0.006 for waitlist group during intervention). CPs reported improved ability to manage stress (*ES* = 0.484, *p* = 0.021; *ES* = 0.742, *p* = 0.002). Improvements in PWCI QOL were mediated by PWCI self‐reported well‐being and CP‐reported stress management. Exploratory analyses suggested a 30% reduction in falls ‘on’ versus ‘off’ the program (not statistically significant in all analyses). No significant differences in other secondary outcomes (e.g., isolation, mobility, cognitive function, burden). No study‐related serious adverse events; only one possibly related AE (low back pain).	**Telehealth (Moving Together)**: 12 weeks (1 h, 2x/week) **Conventional Intervention (waitlist)**: 12 weeks waitlist, then same 12‐week program	Primarily home‐based or any participant‐chosen location with internet access and a suitable device; PWCI and CPs participate together from the same physical location, with U.S. residency required.
Graessel et al., 2024 [58]	**Hardware**: tablet or personal computer with internet access **Software**: MAKSCog individualised computerised cognitive training (iCCT) application (adaptive, machine‐learning based) vs. basic CCT app	Not explicitly classified as telehealth, but the study was conducted entirely virtually, with the intervention being primarily asynchronous (self‐administered home‐based CCT without real‐time provider interaction) and assessments synchronous (via videoconferencing and telephone).	**Telehealth**: Not framed as telehealth, but if interpreted broadly, it's a virtual, multi‐domain cognitive telerehabilitation program (non‐pharmacological, individualised via ML) for MCI, delivered remotely with virtual study procedures. **Conventional intervention**: basic, non‐adaptive CCT	To determine whether individualised CCT (iCCT) results in significantly greater enhancements in overall cognitive functioning for community‐dwelling individuals with MCI (age 60 +) compared to basic CCT (bCCT) over a 6‐month period.	Both groups showed significant improvements in global cognition (MoCA score), but the intervention group (iCCT) had a greater increase (average + 2.2 points, 95% CI [1.4, 2.9], *p* < 0.001) compared to the control group (bCCT, average +0.9 points, 95% CI [0.2, 1.7], *p* = 0.018). The time‐by‐group interaction was significant (F = 4.92, *p* = 0.029, partial *η* ^2^ = 0.057, small to medium effect). Participants used CCTs ∼3 times/week for ∼35 min/session. iCCT was rated more attractive and stimulating. No effects on secondary outcomes like depression or quality of life (reported elsewhere).	6 months.	Primarily home‐based (community‐dwelling), with the entire study conducted virtually; no in‐person components.

**Abbreviations**: AD, Alzheimer's disease; CT, cognitive training; iMCSP, individualised meeting centres support Program; PwD, people with dementia; QoL, quality of life; TR, telerehabilitation; VRRS, virtual reality rehabilitation system.

### 3.2 Outcomes of the Telehealth Interventions

Across all studies, telehealth interventions produced significant improvements in symptoms compared to control groups. Three studies explored the impact of telemedicine on participants’ lifestyles [45, 55, 56]. In the study by Sara Bernin et al., the intervention group exhibited significantly higher cognitive reserve and improved lifestyle routines compared to the control group [45]. Another study demonstrated that a self‐management intervention using eHealth resulted in improvements in both behavioural and biological aspects of dementia, along with increased participation [55]. Additionally, Zuschnegg et al. found that tablet‐based multimodal training resulted in significant improvements in cognitive functioning in patients with Alzheimer's disease [56]. Manenti et al. reported that telerehabilitation using virtual reality led to significant improvements in cognitive abilities (visual‐structural abilities, memory and language) compared to conventional face‐to‐face cognitive therapy [46].

In addition to patient outcomes, several studies reported benefits for caregivers. Six studies aimed to improve the quality of life not only for elderly individuals but also for their caregivers [48, 49, 50, 52, 53, 57]. For example, Torkamani et al. found that the ALADDIN computer platform, which provided dementia‐related educational materials to caregivers, opportunities for interaction with other carers and clinicians and remote monitoring by clinicians, reduced caregiver distress and led to significant improvements in well‐being compared to those in the control group [48]. The FamTechCare intervention, a telehealth support and education program that connected family caregivers to dementia care experts through feedback on video‐recorded care situations, was shown to decrease depression, increase caregiver competence and was reported to be cost‐effective [49]. Another study discovered that caretakers in the intervention group encountered better‐approving health changes [50]. In the study by Rose‐Marie Dröes et al., caregivers in the intervention group reported greater happiness compared to those in the control group [52]. Telehealth was also found to reduce caregiver distress and alleviate the severity of behavioural symptoms [53]. Additionally, Barnes et al. demonstrated that a livestream group movement program improved caregiver well‐being and supported care partners of individuals with cognitive impairment [57]. Finally, one study utilised video conferencing as the primary delivery method for telehealth services [51].

## 4 Discussion

The findings of this systematic review have several key implications for clinical practice. The high success rate of home‐based interventions, which accounted for 57% of the studies, highlights the potential of telehealth to deliver specialised care to patients facing accessibility barriers, particularly those in rural or underserved regions [51]. This is particularly relevant given the increasing demand for quality healthcare services among geographically dispersed populations.

Our review further underscores the significance of flexible delivery methods in telehealth interventions. The comparable effectiveness of synchronous and asynchronous approaches, as well as hybrid models, indicates that healthcare providers can tailor delivery methods to patient preferences and capabilities without compromising treatment outcomes [48, 54, 55, 56, 57, 58]. This adaptability is essential for addressing the diverse needs of patients with Alzheimer's disease and their caregivers, ensuring personalised and effective care across varied settings and technological platforms.

While cost analysis was not a primary focus of most studies included in this review, the cost‐effectiveness of the FamTechCare intervention, along with reductions in travel and facility usage, suggests potential economic advantages for both healthcare systems and patients. This observation aligns with previous research indicating that telehealth can contribute to more cost‐efficient healthcare delivery models [49]. Additionally, the findings from Barnes et al. [57] further support the economic benefits of telehealth by demonstrating the feasibility of a livestream group movement program, which could reduce the need for in‐person sessions while maintaining care quality. These observations align with prior research indicating that telehealth can contribute to more cost‐efficient healthcare delivery models [49, 57].

Additionally, the positive outcomes for both patients and caregivers provide strong support for the development of integrated care models. These models, delivered via telehealth platforms, can address the needs of both groups simultaneously, potentially leading to more holistic and effective care delivery. The findings emphasise the growing relevance of telehealth in managing cognitive impairments, including MCI, dementia and Alzheimer's disease. Across diverse populations and study designs, telehealth interventions consistently showed efficacy in enhancing cognitive function, mobility and caregiver well‐being. Programs that incorporated synchronous and asynchronous modalities, such as VR‐based rehabilitation and eHealth platforms, were not only feasible and effective but also demonstrated good compliance and low attrition rates.

The considerable improvements in quality of life and the reduction in caregiver burden further highlight the potential of telehealth as an effective alternative to in‐person care. However, challenges remain, including small sample sizes in certain studies and issues related to technological accessibility and standardisation. Future research should aim to broaden access and refine protocols to fully optimise the benefits of telehealth in cognitive care.

Future research should focus on several critical areas. One key need is for longer‐term studies, as most current interventions lasted between 6 weeks and 18 months. Conducting extended longitudinal studies would provide deeper insights into the lasting effects of telehealth interventions on both disease progression and caregiver well‐being. Moreover, while a range of technological platforms have proven effective, additional research is necessary to identify which specific features and technologies offer the greatest benefit for different patient subgroups and stages of disease progression. Future research in telehealth for Alzheimer's disease should explore the development of web‐based registry systems to standardise data collection on patient outcomes and caregiver support, as demonstrated in recent multidisciplinary efforts for outpatient rehabilitation [59]. Additionally, game‐based telehealth interventions, such as the Kiddo application, shown to improve ADHD symptoms in children, could be adapted to enhance cognitive engagement and quality of life in Alzheimer's patients, warranting further investigation [60]. Moreover, open access scheduling systems have demonstrated potential in reducing no‐show rates in outpatient clinics, which could be integrated with telehealth platforms to improve patient adherence and access for Alzheimer's care [61]. Furthermore, evaluating the performance of hospital information systems, with a focus on human factors and data security as highlighted by earlier studies, could provide a foundation for enhancing telehealth infrastructure to support Alzheimer's care more effectively [62].

Future research should also prioritise identifying and overcoming implementation barriers, particularly for older adults with limited technological experience. This will be essential for improving adoption rates and ensuring the effective delivery of telehealth services. Furthermore, conducting thorough economic evaluations is vital to compare the cost‐effectiveness of telehealth interventions with traditional care models, providing important data to inform healthcare policy decisions.

Several limitations should be considered when interpreting the results of this systematic review. The included studies varied significantly in terms of intervention methods, outcome measures and follow‐up durations, making direct comparisons difficult. Additionally, the wide range of sample sizes, from 6 to 2724 participants, may impact the generalisability of certain findings. The preponderance of studies from developed countries may also limit the applicability of the results to settings with fewer resources. Finally, it is important to note that the studies generally assumed access to necessary technology and reliable internet connectivity, which may not be universally available to all potential users of telehealth interventions.

Despite these limitations, the positive results observed across different studies and settings suggest that telehealth interventions have great potential to support individuals with Alzheimer's disease and their caregivers. These findings provide a solid foundation for the further development and implementation of telehealth solutions in Alzheimer's care, while also highlighting key areas for future research and improvement.

## 5 Conclusions

The evidence consistently indicated that telehealth interventions, including virtual reality, video conferencing and specialised platforms, resulted in notable improvements compared to traditional care approaches.

The findings highlighted that home‐based interventions, which constituted 73% of the studies, effectively overcame geographical challenges while preserving care quality. Both synchronous and asynchronous delivery methods were found to be effective, with intervention durations ranging from 6 weeks to 18 months, yielding positive outcomes. Additionally, a significant and consistent improvement in caregiver outcomes was observed, particularly in terms of reduced distress and increased caregiving competence.

These findings suggest that telehealth could play a key role in the comprehensive care of Alzheimer's disease, offering an effective alternative or supplement to conventional in‐person care methods. As the prevalence of Alzheimer's disease continues to rise globally, integrating telehealth into standard care practices may provide a scalable solution to meet the growing demand for care, while ensuring accessibility and maintaining the quality‐of‐care service.

## Author Contributions

L.E. and K.G. have designed the study structure and goals. R.M., S.M., M.S.K., F.T.S. and M.V. contribute to data gathering. K.G. and R.M. and S.M. contributed to data gathering and data interpretation. L.E. and K.G. contribute to manuscript preparation. All authors have contributed to finalising and approving the manuscript.

## Funding

The authors have nothing to report.

## Conflicts of Interest

The authors declare that they have no known competing financial interests or personal relationships that could have appeared to influence the work reported in this paper.

## Data Availability

The authors have nothing to report.

## Appendix 1 The JBI checklist.

Question 1: Was true randomisation used for assignment of participants to treatment groups?

□ Yes □ No

Question 2: Was allocation to groups concealed?

□ Yes □ No

Question 3: Were treatment groups similar at the baseline?

□ Yes □ No

Question 4: Were participants blind to treatment assignment?

□ Yes □ No

Question 5: Were those delivering the treatment blind to treatment assignment?

□ Yes □ No

Question 6: Were treatment groups treated identically, other than the intervention of interest?

□ Yes □ No

Question 7: Were outcome assessors blind to treatment assignment?

□ Yes □ No

Question 8: Were outcomes measured in the same way for treatment groups?

□ Yes □ No

Question 9: Were outcomes measured in a reliable way?

□ Yes □ No

Question 10: Was follow up complete and if not, were differences between groups in terms of their follow‐up adequately described and analysed?

□ Yes □ No

Question 11: Were participants analysed in the groups to which they were randomised?

□ Yes □ No

Question 12: Was appropriate statistical analysis used?

□ Yes □ No

Question 13: Was the trial design appropriate and any deviations from the standard RCT design (individual randomisation, parallel groups) accounted for in the conduct and analysis of the trial?

□ Yes □ No
